# A Microbial-Based Biostimulant Enhances Sweet Pepper Performance by Metabolic Reprogramming of Phytohormone Profile and Secondary Metabolism

**DOI:** 10.3389/fpls.2020.567388

**Published:** 2020-11-05

**Authors:** Paolo Bonini, Youssef Rouphael, Begoña Miras-Moreno, Byungha Lee, Mariateresa Cardarelli, Gorka Erice, Veronica Cirino, Luigi Lucini, Giuseppe Colla

**Affiliations:** ^1^Next Generation Agronomics Laboratory (NGAlab), La Riera de Gaia, Tarragona, Spain; ^2^Department of Agricultural Sciences, University of Naples Federico II, Portici, Italy; ^3^Department for Sustainable Food Process, Research Centre for Nutrigenomics and Proteomics, Università Cattolica del Sacro Cuore, Piacenza, Italy; ^4^Consiglio per la ricerca in agricoltura e l’analisi dell’economia agraria, Centro di ricerca Orticoltura e Florovivaismo, Pontecagnano Faiano, Italy; ^5^Atens, La Riera de Gaia, Tarragona, Spain; ^6^Department of Agriculture and Forest Sciences, Università degli Studi della Tuscia, Viterbo, Italy

**Keywords:** *Funneliformis mosseae*, *Rhizoglomus irregularis*, *Trichoderma koningii*, *Capsicum annuum* L., plant metabolomics, metabolic reprogramming

## Abstract

Microbial-based biostimulants can improve crop productivity by modulating cell metabolic pathways including hormonal balance. However, little is known about the microbial-mediated molecular changes causing yield increase. The present study elucidates the metabolomic modulation occurring in pepper (*Capsicum annuum* L.) leaves at the vegetative and reproductive phenological stages, in response to microbial-based biostimulants. The arbuscular mycorrhizal fungi *Rhizoglomus irregularis* and *Funneliformis mosseae*, as well as *Trichoderma koningii*, were used in this work. The application of endophytic fungi significantly increased total fruit yield by 23.7% compared to that of untreated plants. Multivariate statistics indicated that the biostimulant treatment substantially altered the shape of the metabolic profile of pepper. Compared to the untreated control, the plants treated with microbial biostimulants presented with modified gibberellin, auxin, and cytokinin patterns. The biostimulant treatment also induced secondary metabolism and caused carotenoids, saponins, and phenolic compounds to accumulate in the plants. Differential metabolomic signatures indicated diverse and concerted biochemical responses in the plants following the colonization of their roots by beneficial microorganisms. The above findings demonstrated a clear link between microbial-mediated yield increase and a strong up-regulation of hormonal and secondary metabolic pathways associated with growth stimulation and crop defense to environmental stresses.

## Introduction

Three major current global challenges are food security, environmental degradation, and climate change. The first may be augmented, and the latter two diminished by improving nutrient (nitrogen, phosphorus) use efficiency in agricultural crop production and stabilizing yield by practicing sustainable agriculture (Searchinger et al., 2018). The application of plant biostimulants such as beneficial microbes [arbuscular mycorrhizal fungi (AMF), *Trichoderma* spp., plant growth-promoting rhizobacteria (PGPR)], and bioactive substances (humic and fulvic acids, macroalgae and microalgae, protein hydrolysates and silicon) used either separately or in combination may help crops contend with the challenges mentioned above (Rouphael and Colla, 2020).

Plant biostimulants were recently defined in the Regulations of the European Parliament and Council (Regulation EU 2019/1009) as “…*EU fertilising product(s) able to stimulate plant nutrition processes independently of the product’s nutrient content with the sole aim of improving one or more of the following characteristics of the plant or the plant rhizosphere: (1) nutrient use efficiency, (2) tolerance to abiotic stress, (3) quality traits, or (4) availability of confined nutrients in the soil or rhizosphere*”. AMF comprise a very important category of biostimulants (Rouphael et al., 2015; Bitterlich et al., 2018). They are members of the *Glomeromycotina* subphylum and establish mutualistic relationships with 74% of all terrestrial plant species (Spatafora et al., 2016). AMF boost productivity and enhance tolerance to abiotic stress (high temperature, drought, and salinity) in crops (Rouphael et al., 2015). These findings are due to the AMF-mediated enhancement of (1) growth and vigor of the root apparatus in terms of biomass, length, density, and branching; (2) macronutrient (N, P, and Fe) and micronutrient (Mn and Zn) uptake and assimilation; (3) water relations and photosynthetic activity; (4) secondary metabolism; (5) release of low- and high-molecular-weight organic compounds such as amino acids, phenolics, organic acids, and proteins into the rhizosphere; (6) phytohormone signaling (Rouphael et al., 2015, 2020b; Yakhin et al., 2017; Rouphael and Colla, 2018). The indirect and direct mechanisms of AMF influence shoot and root function and augment crop agronomic performance. Other plant beneficial endophytic fungi include *Trichoderma* spp. Several of them are registered as microbial biological control agents (López-Bucio et al., 2015; Rouphael et al., 2020a). However, several studies reported that certain *Trichoderma* spp. including *T. atroviride*, *T. koningii*, *T. harzianum*, and *T. virens* are other plant biostimulants that boost crop performance (Colla et al., 2015) and nutrient use efficiency and/or endue plants with abiotic stress tolerance (Saia et al., 2020). The direct and indirect mechanisms of the biostimulant action of *Trichoderma* strains include (i) improvement of lateral root development, (ii) induction of plant mitogen-activated protein 6, and (iii) production and rhizosphere excretion of auxins and secondary metabolites such as volatile and non-volatile substances that stimulate various plant responses and enhance crop nutrient uptake, resilience, and productivity (López-Bucio et al., 2015).

The beneficial effects of combinations of AMF and *Trichoderma* on vegetable crops were previously demonstrated under both optimal and suboptimal conditions (Colla et al., 2015; Saia et al., 2020). However, the physiological and molecular mechanisms underlining biostimulant action have not been fully elucidated. One strategy to clarify biostimulant efficacy is to analyze metabolic profiling. In turn, this process serves as a basis for subsequent transcriptomic analyses. The metabolomic phytochemical characterization could identify numerous physiological processes and metabolic pathways modulated by biostimulants (Yakhin et al., 2017). The above approach has been never used in an important vegetable crop such as pepper (*Capsicum* spp.) where biostimulant applications (e.g., vegetal-derived substances, arbuscular mycorrhizal fungi, plant growth-promoting microorganisms) have proven to be beneficial in ameliorating the growth, yield and nutritional value of fruits (Ertani et al., 2014; Pereira et al., 2016).

It has been hypothesized that AMF and *Trichoderma* can induce and enhance fruit yield by modulating the hormonal balance and secondary metabolic pathways.

In the present study, then, an untargeted metabolomics approach was conducted on greenhouse pepper. The objectives were to illuminate metabolomic reprogramming by microbial biostimulants in leaf tissue at the vegetative and reproductive phenological stages, elucidate biostimulant regulation of key phytohormones, and correlate these molecular-level biostimulant-promoted changes to observed fruit yield and quality variations.

## Materials and Methods

### Growth Conditions, Plant Material, Crop Management, and Experimental Design

The trial was conducted in a greenhouse located at Paraje Águilas Bajas, Santa María del Águila, Almería, Spain (36°47′39″N 2°46′32″W). The greenhouse was composed of polycarbonate walls and a roof made of tri-laminated low-density polyethylene (LDPE) film (200 μm thickness) with ∼60% spectral transmittance in the photosynthetically active radiation (PAR) region. The greenhouse was unheated and passively ventilated with lateral side panels and flap roof windows. It had an east-west orientation and a north-south crop row alignment. The air temperature and relative humidity inside the greenhouse were in the ranges of 12–32 °C and 50–70%, respectively. Transplants of the sweet pepper (*Capsicum annuum* L.) hybrid ‘SV1204PB’ (Seminis, Montornés del Vallés, Barcelona, Spain) at the 4–5 true-leaf stage were planted in “Enarenado” sandy soil commonly used in greenhouse production in Almería. This soil is formed by placing a 20 cm layer of sandy loam soil, imported from a quarry, over the original stony, loam soil. A 10 cm layer of coarse river sand is placed over the imported sandy loam soil as a mulch (Thompson et al., 2007). The planting date was 19 July 2017, and the planting density was 2.0 m^–2^. The soil composition was 13.5% (w/w) clay, 72.8% (w/w) sand, and 13.7% (w/w) silt. Soil pH was 7.52, with an organic matter content of 0.71%, and total nitrogen, available phosphorus, and exchangeable potassium of 690, 51.4, and 321 mg kg^–1^, respectively. Aerial drip irrigation was used. The in-line emitters were positioned at 0.30 m intervals, and the emitter flow rate was 3.4 L h^–1^. Preplant fertilizer was broadcast at 90 kg ⋅ ha^–1^ P, 120 kg ⋅ ha^–1^ K, and 15 kg ⋅ ha^–1^ Mg and incorporated into the soil. Additional fertilizer in the form of K_2_SO_4_ (80 kg ⋅ ha^–1^ K) was applied through the drip irrigation system. Nitrogen was applied via fertigation in the form of 27% NH_4_NO_3_ soluble fertilizer starting 10 days after transplanting until day 83. The total N supply was split into 10 weekly dressings. Powdery mildew caused by *Leveillula taurica* was controlled by three foliar applications of penconazole (Topas 10EC; Syngenta, Madrid, Spain) at the label-recommended rate. Aphids and spider mites were controlled by one foliar application each of imidacloprid (Confidor 200 SL; Bayer Crop Science, Valencia, Spain) and fenpyroximate (Miro; Bayer Crop Science, Valencia, Spain), respectively. Weeds were controlled by hand hoeing. The control and microbial-based biostimulant treatments were compared in a randomized block design with four replicates for a total of eight experimental plots. Each experimental plot was 30 m^2^ and contained 60 plants in four single rows. The microbial-based biostimulants were applied through a drip irrigation system. The first application was made at 15 days after transplanting (DAT) (3 August 2017) at the rates of 1 × 10^6^ spores ha^–1^ *Rhizoglomus irregularis* BEG72 and 1 × 10^6^ spores ha^–1^ *Funneliformis mosseae* BEG234 in the form of 2.0 kg ha^–1^ Team Horticola (Agrotecnologías Naturales, S.L., Tarragona, Spain) plus 1 × 10^12^ CFU ha^–1^ *Trichoderma koningii* TK7 in the form of 1.0 kg ha^–1^ Condor Shield (Agrotecnologías Naturales, S.L., Tarragona, Spain). The second treatment was applied 43 DAT (31 August 2017) at the rate of 5 × 10^11^ CFU ha^–1^ *Trichoderma koningii* TK7 as 0.5 kg Condor Shield (Agrotecnologías Naturales, S.L., Tarragona, Spain). Multiple applications of *Trichoderma* inoculum are recommended especially in long-term crops like greenhouse pepper under soils with low organic matter to raise the population of this saprophytic beneficial fungus in the soil rhizosphere. Because arbuscular mycorrhizal fungi such as *Rhizoglomus irregularis* and *Funneliformis mosseae* are symbiotic microorganisms, it is usually sufficient the application of mycorrhizal inoculum just once at the beginning of cropping cycle (Colla et al., 2008).

### Yield Measurements and Arbuscular Mycorrhizal Fungi (AMF) Root Colonization

Fully mature pepper fruits were harvested from 139 DAT (5 December 2017) to 272 DAT (17 April 2018) on 26 plants per each plot. Mean fruit weight and number and marketable yield were determined for each experimental plot (replicate). Rotten fruit and those weighing < 100 g were considered unmarketable yield.

At the end of the trial, the roots of six pepper plants per experimental plot were rinsed, and subsamples were used to evaluate AMF root colonization. The root samples were cleared with 10% (w/v) KOH, stained with 0.05% (w/v) trypan blue in lactophenol, and microscopically (Stereo microscope Leica EZ4V, 32x—Leica Microsystems Srl, Buccinasco, Italy) examined for AMF colonization. The percentage of colonized root segments was determined by the grid line intersect method (Giovannetti and Mosse, 1980).

### Quantitative Real-Time PCR (qPCR) for Determining Concentration of Strain TK7 in Soil

At the end of the trial, rhizosphere soil was collected by shaking the roots collected from 10 plants per plot. The concentration of *T. koningii* TK7 in the rhizosphere was determined using a qPCR approach with two strain-specific primers, named RM3 (GGAGGCTTGAATGGGA) and RM4 (CAAAACGCTGCTAAGG), targeting to a coding sequence annotated as hypothetical protein. The DNA template used in qPCR experiments was extracted from the soil samples with a DNeasy^®^ Powersoil^®^ kit (Cat. No. 12888-50; Qiagen, Hilden, Germany) according to Qiacube (Qiagen, Hilden, Germany) automation procedures. Amplification reactions were carried out in a 20 μL final volume on a Rotor-Gene Q apparatus (Qiagen, Hilden, Germany). Reactions contained: 4 μL of DNA sample;10 μL of QuaniNova^TM^ SYBR^®^ Green Supermix (2x); 0.14 μL of 25 μM primers; 4.72 10-μL of water. The qPCR cycling conditions were as follows: initial incubation at 95°C for 2 min, 45 cycles of 95°C for 5 s each, and 60°C for 12 s. Two technical replicates were performed per sample. After qPCR, the number of colony forming unit (CFU) equivalent per gram of soil was calculated by interpolation of calibration curves obtained using serial dilutions (1:1,000, 1:10,000, and 1:100,000) of a DNA preparation extracted from 10^9^ CFU mL^–1^ culture aliquots of the target strain.

### Sample Collection and Untargeted Metabolomics

Four leaves in the third position from the branch tip were harvested for untargeted metabolomics at 43 DAT (31 August 2017) and at 131 DAT (27 November 2017). The leaves were flash-frozen in liquid nitrogen and stored at −80 °C until subsequent metabolomic analysis.

The four leaves from each replicate were pooled and homogenized, then 1.0 g was extracted in 0.1% HCOOH in 80% methanol using an ultra-turrax, as previously described (Paul et al., 2019). An untargeted metabolomics approach was conducted in the UHPLC 1290 chromatographic system coupled to a hybrid quadrupole-time-of-flight (Q-TOF) G6550 mass spectrometer (UHPLC/Q-TOF) (Agilent Technologies, Santa Clara, CA, United States). A Waters Acquity UPLC^®^ BEH C18 column (100 × 2.1 mm i.d., 1.7 μm) (Waters Corp., Milford, MA, United States) was used for reverse-phase chromatographic separation. The binary gradient consisted of water and acetonitrile and the Riken Plasma method was followed (Tsugawa et al., 2019). The injection volume was 2 μL and the mass spectrometer was run in positive polarity and SCAN mode (range: 100–1,700 m/z; extended dynamic range setting). Quality controls (QC) were prepared by pooling 10 μL samples. Five QCs were acquired in data-dependent mode (auto MS/MS) at 1 Hz, 10 precursors/cycle, collision energies of 10 V, 30 V, and 50 V), and in iterative mode with active exclusion to increase the number of compounds targeted for tandem MS fragmentation.

Alignment, blank filtration, and identification were performed in MSDIAL v. 4.0 (Riken, Tokyo, Japan) using the publicly available library MoNA (Mass Bank of North America) and an internal standard compound library as specified in the Supplementary Table 1. Compounds lacking experimental MS/MS spectra were annotated with MSFINDER (Riken, Tokyo, Japan) following the procedure described in Blaženović et al. (2019). The alternatives were filtered by retention time prediction (Bonini et al., 2020). MSI (metabolomics standards initiative) levels for each identified compound are listed in Supplementary Table 1.

### Statistics and Data Analysis

Data were statistically analyzed with SPSS v. 21 (IBM Corp., Armonk, NY, United States). The microbial-based biostimulant effects on mycorrhizal root colonization, *Trichoderma* population, fruit yield and yield components were analyzed by an unpaired Student’s *t*-test. A *p*-value of less than or equal to 0.05 was considered to indicate significant difference. Values are presented as means plus/minus standard deviation.

Concerning metabolomics, the compound intensity table exported from MSDIAL v. 4.0 (Riken, Tokyo, Japan) (Tsugawa et al., 2015) was uploaded into MS-FLO (Riken, Tokyo, Japan) (De Felice et al., 2017) to reduce false positives and duplicates. The output was then imported into R v. 3.6.0 for centring (normalization against the median), scaling, PCA, and calculation of fold changes, ANOVA (Benjamini-Hochberg FDR multiple testing correction, *P* < 0.05). Venn diagrams were plotted to identify metabolites common to 43 and 131 DAT sampling points but not exclusive to a particular growth stage. Compounds with *P* < 0.05 were imported into ChemRICH (Barupal and Fiehn, 2017) for enrichment analysis based on their chemical similarity and MetaMapp (Barupal et al., 2012) for chemical network analysis. Cytoscape (Saito et al., 2012) displayed exported MetaMapp data and plotted the final images.

## Results

### Soil Fungal Concentration and Crop Yield

By the end of the trial, the percentage of mycorrhizal root colonization was significantly (*P* < 0.01) higher under the microbial inoculation treatment (33.6 ± 11.7%) than it was under the uninoculated control treatment (8.0 ± 4.9%). The total number of *Trichoderma* colonies estimated by qPCR in the rhizosphere of inoculated pepper plants was significantly (*P* < 0.01) higher than that recorded for the untreated control (2.2 × 10^5^ ± 0.6 × 10^5^ vs. 1.2 × 10^3^ ± 0.4 × 10^3^ CFU g^–1^, respectively). It is worth mentioning that the weak PCR amplification signal observed in control experiments with metagenome from not inoculated soil did not interfere with the quantitative PCR analysis.

Relative to the uninoculated control, inoculation with AMF and *Trichoderma koningii* significantly increased fruit yield at single harvests (139, 174, 272 DAT) and as a total (Table 1); moreover, the biostimulant-mediated yield increase was more pronounced during the first part of the reproductive cycle, namely, early yield (139 and 174 DAT) (Table 1). The comparatively higher production rates measured at 139 DAT and 272 DAT for pepper plants inoculated with microbial-based biostimulant was due to an increase in mean fruit weight. In contrast, the relatively higher fruit yield determined for 174 DAT was attributed to increases in both fruit number per plant and mean fruit mass (Tables 1–3). The microbial-based biostimulant significantly improved cumulative fruit yield by an average of 23.7% relative to uninoculated pepper plants (Table 1).

**TABLE 1:** Effect of microbial-based biostimulant application on fruit yield of greenhouse-grown peppers at different days after transplanting (DAT).

Treatment	Fruit yield (kg plant^–1^)					
	139 DAT	174 DAT	243 DAT	264 DAT	272 DAT	Total
	**139 DAT**	**174 DAT**	**243 DAT**	**264 DAT**	**272 DAT**	**Total**
Control	0.57 ± 0.04	0.85 ± 0.07	0.59 ± 0.09	0.73 ± 0.06	0.63 ± 0.13	3.37 ± 0.07
Biostimulant	0.73 ± 0.10	1.41 ± 0.30	0.48 ± 0.13	0.74 ± 0.07	0.81 ± 0.06	4.17 ± 0.07
Significance	*	**	Ns	Ns	*	**

Mean values plus/minus standard deviations (n = 4); Two-tailed unpaired Student’s t-test, ns = not significant, *P < 0.05, and **P < 0.01.

**TABLE 2:** Effect of microbial-based biostimulant application on fruit number of greenhouse-grown peppers at different days after transplanting (DAT).

Treatment	Fruit number (n. plant^–1^)					
	139 DAT	174 DAT	243 DAT	264 DAT	272 DAT	Total
Control	2.35 ± 0.37	3.65 ± 0.29	2.90 ± 0.53	2.20 ± 0.10	2.09 ± 0.45	13.20 ± 0.50
Biostimulant	2.75 ± 0.22	5.56 ± 1.00	1.92 ± 0.67	1.98 ± 0.20	2.42 ± 0.11	14.62 ± 0.83
Significance	ns	**	Ns	Ns	ns	*

Mean values plus/minus standard deviations (n = 4); Two-tailed unpaired Student’s t-test, ns = not significant, *P < 0.05, and **P < 0.01.

**TABLE 3:** Effect of microbial-based biostimulant application on fruit mean weight of greenhouse-grown peppers at different days after transplanting (DAT).

Treatment	Fruit mean weight (g fruit^–1^)				
	139 DAT	174 DAT	243 DAT	264 DAT	272 DAT
Control	244.0 ± 5.8	232.4 ± 2.4	204.4 ± 27.6	335.1 ± 24.1	302.5 ± 20.3
Biostimulant	264.7 ± 7.9	254.4 ± 12.8	250.4 ± 27.5	368.3 ± 3.5	334.3 ± 15.4
Significance	**	**	Ns	*	*

Mean values plus/minus standard deviations (n = 4); Two-tailed unpaired Student’s t-test, ns = not significant, *P < 0.05, and **P < 0.01.

### Yields and Modulation of Metabolomic Profile

**FIGURE 1:**
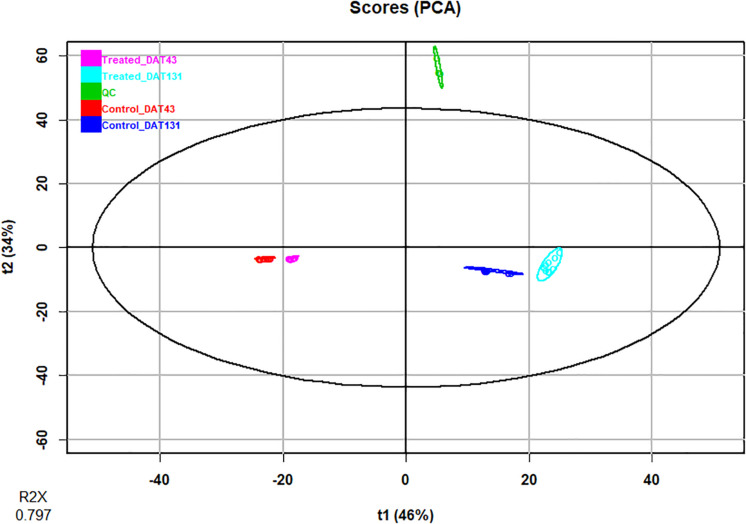
Principal Component Analysis (PCA) of identified metabolites in pepper plants following treatment with microbial biostimulants. Compounds were profiled by untargeted metabolomics and samples harvested at two sampling dates: 43 (vegetative stage), and 131 days after transplanting (reproductive stage).

**FIGURE 2:**
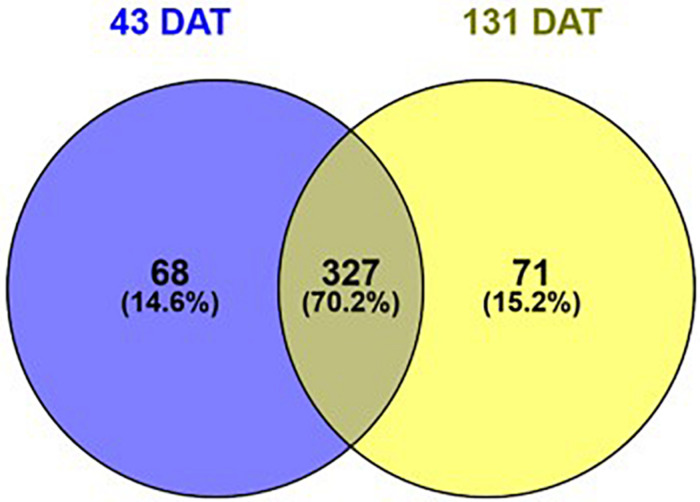
Venn diagram of statistically different metabolites (*P* < 0.05) in pepper plants following treatment with microbial biostimulants, as a function of the sampling date. Compounds were profiled by untargeted metabolomics at two sampling dates: 43 (vegetative stage), and 131 days after transplanting (reproductive stage).

In the present study, we inoculated pepper plants with the AMF species *Rhizoglomus irregularis* and *Funneliformis mosseae* and *Trichoderma koningii*. Microbial treatments accelerated and increased total crop yield by 24%, relative to uninoculated plants (Table 1). Such increase in pepper yield was attributed to the gain in fruit weight and/or number. Ultra-high-performance liquid chromatography quadrupole-time-of-flight high-resolution mass spectrometry (UHPLC-QTOF) and annotation in publicly available databases and large metabolite groups were conducted to obtain wide metabolome coverage. We applied UHPLC-QTOF-based untargeted metabolomic profiling of crude extracts to assess relative differences in the vegetative stage (43 DAT) and reproductive stage (131 DAT) leaf metabolite profiles between inoculated and uninoculated plants. A principal component analysis (PCA) explained 79% of the overall variance. The PCA score plot (Figure 1) showed two main clusters accounting for the vegetative and reproductive stages, respectively. Within each cluster, the metabolomic profiles of leaves from inoculated and those from the uninoculated (control) plants did not show overlapping, thus indicating distinct phytochemical signatures. Notably, considering that PCA provides unsupervised descriptions of relatedness/unrelatedness across treatments, these patterns indicate a metabolomic shift in plants following the biostimulant treatments. Thereafter, *t*-test ANOVA (*P* < 0.01) was carried out to identify differentially accumulated metabolites at each plant growth stage. This analysis disclosed > 466 annotated metabolites (Sheets 2 and 3 of Supplementary File 1) that had significantly changed between the vegetative and reproductive stages. Of these, 327 were common to 43 and 131 DAT sampling points (Figure 2). In contrast, 68 and 71 metabolites differentially accumulated during the vegetative (43 DAT) and reproductive (131 DAT) stages, respectively (Figure 2). The interactions between microbial inoculants and plants are complex. Nevertheless, metabolomics effectively included the metabolic responses and mechanisms involved in the plant-microbe interactions. Considering that 327 common metabolites (i.e., 70%) out of 395 and 398 metabolites (*P* < 0.05) at 43 and 131 DAT, respectively, were shared between vegetative and reproductive phenological stages (Figure 2), the biostimulant-mediated metabolomic shifts we recorded represented a common signature, irrespective from the plant growth stage. On the other hand, certain stage-specific responses could be identified as well.

**FIGURE 3:**
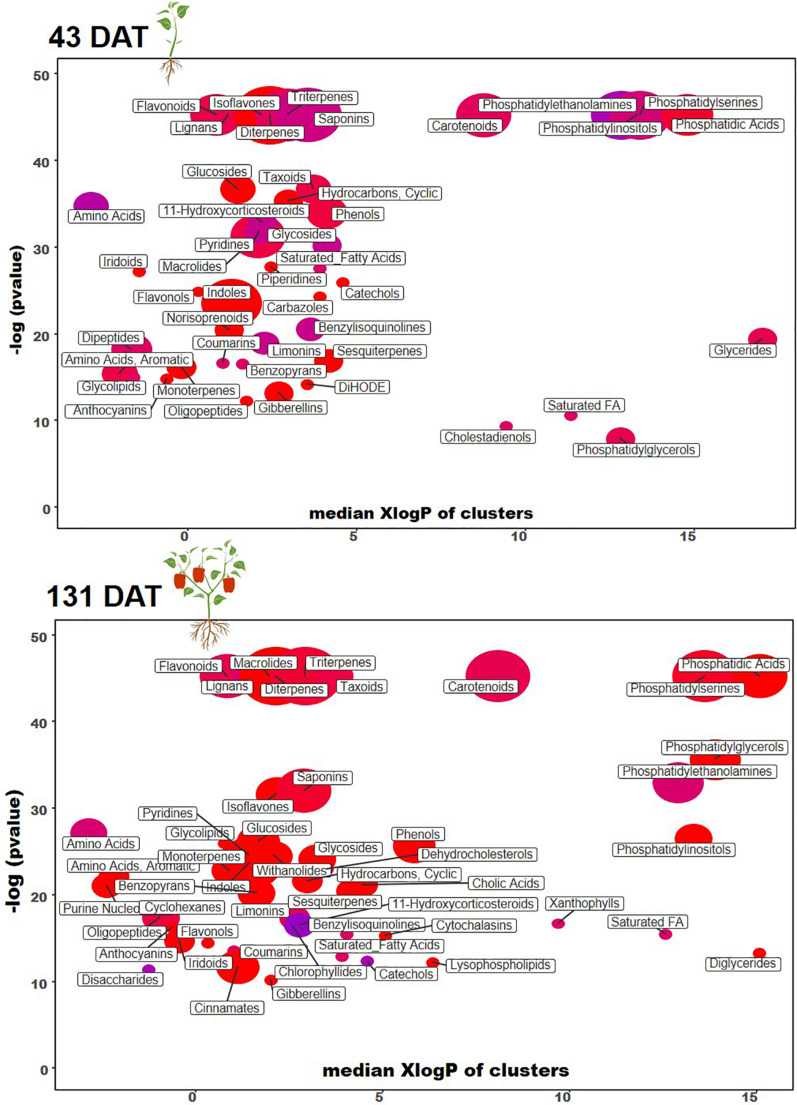
Chemical similarity enrichment analysis (ChemRICH) of statistically different annotated metabolites in microbial-based biostimulant treated leaves compared to untreated control at 43 (vegetative stage) and 131 days after transplanting (reproductive stage). Color is according to proportion of increased or decreased compounds (red = increased, blue = decreased, pink = mixed) within each cluster.

**FIGURE 4:**
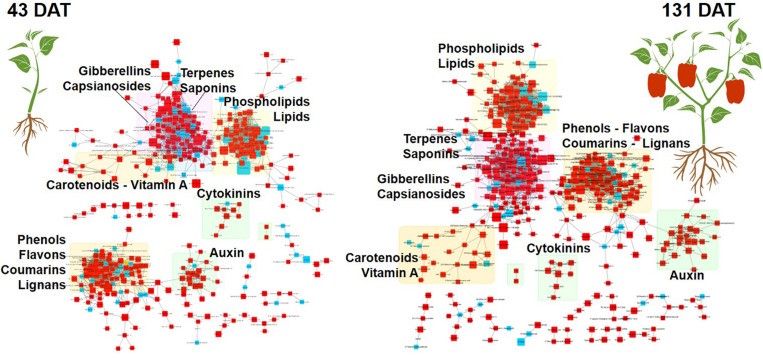
MetaMapp Metabolomic network maps of pepper leaves at 43 (vegetative stage) and 131 days after transplanting (reproductive stage). Microbial-based biostimulant treated plants were compared to control ones. The red squares are compounds with an increase in fold change, while the blue ones represent compounds with a decrease in fold changes. Chemical similarity and KEGG reaction were utilized to draw the clusters and nodes.

To clarify and visualize the variations between metabolic profiles at the vegetative and reproductive stages, we performed a chemical enrichment analysis using ChemRICH (Figure 3 and Tables 4, 5) and plotted the output by MetaMapp Cytoscape (Figure 4; Barupal and Fiehn, 2017). Most of the significantly upregulated and downregulated metabolites (fold-change values ≤ 0.5 and ≥ 1.5, respectively; *P* ≤ 0.01) had a wide range of functions including growth stimulation, antifungal activity, pathogen resistance, energy sources, and secondary signaling cofactors.

**TABLE 4:** Effect of microbial-based biostimulant application on compound chemical classes (CHEMRICH) of greenhouse-grown peppers at vegetative stage (43 DAT).

Cluster name	Cluster size	*p*-values	FDR	Key compound	Increased	Decreased
Carotenoids	10	2.2E-20	9.2E-20	Fucoxanthinol, Vit A, Alpha Carotene	7	3
Diterpenes	17	2.2E-20	9.2E-20	NCGC00385284-01_C32H54O13	17	0
Flavonoids	9	2.2E-20	9.2E-20	Skullcapflavone I 2′-(2″-E-cinnamoylglucoside)	7	2
Isoflavones	6	2.2E-20	9.2E-20	Genistein	6	0
Lignans	4	2.2E-20	9.2E-20	Myricatomentoside I	3	1
Phosphatidic Acids	9	2.2E-20	9.2E-20	PA(18:0/18:2)	8	1
Phosphatidylethanolamines	12	2.2E-20	9.2E-20	PE(P-16:0/20:5)	4	8
Phosphatidylinositols	5	2.2E-20	9.2E-20	PIM4(18:1/14:0)	5	0
Phosphatidylserines	12	2.2E-20	9.2E-20	PS(P-16:0/13:0)	7	5
Saponins	15	2.2E-20	9.2E-20	Borassoside A	8	7
Triterpenes	14	2.2E-20	9.2E-20	Cussoracoside F	10	4
Glucosides	5	1.1E-16	3.9E-16	Luteolin-4′-O-glucoside	5	0
Amino Acids	5	7.8E-16	2.2E-15	Arginine	2	3
Phenols	6	1.7E-15	4.5E-15	Gibbilimbol B	5	1
Glycosides	4	7.7E-14	1.7E-13	Melissoidesin D	2	2
Macrolides	10	2.6E-14	5.9E-14	Capsianoside	8	2
Piperidines	3	9.1E-13	1.9E-12	Andrachcinidine	3	0
Iridoids	3	1.5E-12	3E-12	NCGC00168877-02_C15H20O8	3	0
Catechols	3	5.4E-12	9.9E-12	(S)-[8]-Gingerol	3	0
Flavonols	3	1.6E-11	2.7E-11	Kaempferol	3	0
Auxins	12	6E-11	9.8E-11	Indole-3-acetamide	12	0
Glycerides	4	3.7E-09	5.4E-09	DG(19:1(9Z)/22:4(7Z,10Z,13Z,16Z)/0:0)[iso2]	3	1
Limonins	4	5.9E-09	8.4E-09	11beta-Acetoxydihydrocedrelone	2	2
Dipeptides	6	1.2E-08	1.7E-08	Ala-Phe	4	2
Sesquiterpenes	4	4.5E-08	6.1E-08	Leucascandrolide A	4	0
Coumarins	3	5.8E-08	7.6E-08	Coumarin	2	1
Monoterpenes	4	9.6E-08	1.2E-07	NCGC00384740-01_C21H34O9	4	0
Amino Acids, Aromatic	5	0.0000002	2.5E-07	Tryptophan	4	1
Glycolipids	3	3.1E-07	3.6E-07	Lyciumoside IV	2	1
Anthocyanins	3	3.7E-07	4.3E-07	Cyanidine-3-O-sambubioside	3	0
DiHODE	3	6.8E-07	7.6E-07	8(R)-Hydroperoxylinoleic acid	3	0
Gibberellins	4	0.0000019	0.0000021	Gibberellin A20	4	0
Oligopeptides	3	0.0000047	0.000005	Indole-3-acetyl-L-isoleucine	3	0
Saturated FA	3	0.000024	0.000025	Capric acid	2	1
Phosphatidylglycerols	4	0.00037	0.00037	PG(18:2/13:0)	3	1

**TABLE 5:** Effect of microbial-based biostimulant application on compound chemical classes (CHEMRICH) of greenhouse-grown peppers at reproductive stage (131 DAT).

Cluster name	Cluster size	*p*-values	FDR	Key compound	Increased	Decreased
Carotenoids	12	2.2E-20	1.3E-19	Fucoxanthinol, Vit A, Alpha Carotene	9	3
Diterpenes	15	2.2E-20	1.3E-19	Traversianal	15	0
Flavonoids	9	2.2E-20	1.3E-19	Skullcapflavone I 2′-(2″-E-cinnamoylglucoside)	7	2
Lignans	5	2.2E-20	1.3E-19	Myricatomentoside I	3	2
Macrolides	9	2.2E-20	1.3E-19	Capsianoside	8	1
Phosphatidic Acids	9	2.2E-20	1.3E-19	PA(O-18:020:3(8Z11Z14Z))	9	0
Phosphatidylserines	12	2.2E-20	1.3E-19	PS(P-16:013:0)	11	1
Triterpenes	15	2.2E-20	1.3E-19	Tricalysioside T	13	2
Phosphatidylglycerols	8	3.3E-16	1.7E-15	PG(P-18:017:2(9Z12Z))	8	0
Phosphatidylethanolamines	8	4.9E-15	2.1E-14	PE(P-16:020:5(5Z8Z11Z14Z17Z))	5	3
Saponins	9	1.2E-14	4.9E-14	Namonin E	8	1
Isoflavones	6	1.8E-14	6.8E-14	Genistein	6	0
Amino Acids	5	1.6E-12	5.4E-12	L-Valine	3	2
Phosphatidylinositols	5	3.1E-12	1E-11	PIM4(18:1(9Z)14:0)	5	0
Glucosides	6	4.3E-12	1.3E-11	Daedaleaside D	6	0
Glycolipids	3	5.3E-12	1.5E-11	Capsoside A	3	0
Phenols	6	7E-12	1.9E-11	Gibbilimbol B	6	0
Glycosides	5	3.2E-11	7.6E-11	Cyclopassifloside VII	5	0
Auxins	12	5.1E-11	1.2E-10	INDOLE-3-PYRUVIC ACID	12	0
Monoterpenes	5	1.2E-10	2.5E-10	beta-Thujaplicin	5	0
Amino Acids, Aromatic	4	2.3E-10	4.6E-10	34-Dihydroxy-L-phenylalanine	4	0
Purine Nucleosides	4	6.9E-10	1.2E-09	Adenosine	4	0
Sesquiterpenes	4	1.4E-09	2.4E-09	(+)-vulgraon B	4	0
Oligopeptides	5	2.7E-08	4.3E-08	Indole-3-acetyl-L-isoleucine	4	1
Limonins	4	2.9E-08	4.4E-08	Toonaciliatin D	3	1
Xanthophylls	3	5.6E-08	8.3E-08	Spirilloxanthin	2	1
Chlorophyllides	3	8.2E-08	1.2E-07	chlorophyllide a	3	0
Anthocyanins	3	8.4E-08	1.2E-07	Delphinidin-3-O-sambubioside	3	0
Saturated FA	3	1.8E-07	2.5E-07	Petroformyne 1	2	1
Iridoids	4	4.1E-07	5.1E-07	Eleganoside B	4	0
Flavonols	3	5E-07	6.1E-07	Kaempferol	3	0
Coumarins	3	1.3E-06	1.5E-06	Coumarin	2	1
Diglycerides	3	1.7E-06	0.000002	DG(15:1(9Z)22:6(4Z7Z10Z13Z16Z19Z)0:0)iso2	3	0
Saturated_Fatty Acids	3	2.5E-06	2.8E-06	Capric acid	2	1
Catechols	3	4.3E-06	4.8E-06	33′44′-Tetrahydroxy-55′-diisopropyl-22′-dimethylbiphenyl	1	2
Lysophospholipids	3	4.9E-06	5.2E-06	PC(O-17:00:0)	3	0
Cinnamates	6	8.2E-06	8.5E-06	Sinapine	6	0
Disaccharides	3	0.000011	0.000011	Melibiose	1	2
Gibberellins	3	0.000036	0.000036	Gibberellin A20	3	0

Among other, secondary metabolites such as carotenoids and other terpenes, saponins, and phenolic compounds, were altered by the biostimulant treatment. Compared to the control, at 131 DAT, foliar vitamin A and α-carotene were 1.5 × and 8.5 × higher, respectively, following treatment. Blumenols, a class of apocarotenoids or cyclohexanone derivatives of carotenoid cleavage, also accumulated in the biostimulant-treated plants. In detail, blumenol B was 2 × and 2.5 × higher at 43 and 131 DAT, respectively, after biostimulant application. Regarding foliar saponins, their abundance was 1.5–10 × higher in plants treated with biostimulant than in the untreated control. Furthermore, irrespective of growth stage, the phenolics skullcapflavone I, pelargonidin-3-*O*-glucoside, kaempferol, genistein, apiin, and myricatomentoside I accumulated to levels 3–87 × higher in the biostimulant-treated plants than the control.

Phospholipids were also modulated by the treatment. In more detail, the accumulation of phosphatidylethanolamines [PE(P-16:0/20:5)], phosphatidic acid [PA(15:0/22:6), PA(O-18:020:3)], phosphatidylinositol [PIM4(18:1/14:0)], and phosphatidylserine [PS(P-16:013:0)] by 1.5–30 × was recorded in biostimulant-treated plants, compared to the control. Furthermore, lysophospholipids [PA(P-16:0e18:2)] increased by 6.5 × in biostimulant-treated leaves at 131 DAT sampling (reproductive stage).

Concerning hormones, the microbial-based treatment induced also the accumulation of auxins (indole-3-acetamide and indole-3-pyruvic acid increased by 1.7–7.5 × relative to the control), whereas a set of gibberellins precursors (GA81, GA36, GA37, GA12, and GA20) increased by 1.3–16 × compared to control, at both 43 and 131 DAT. Still regarding phytohormones, the biostimulant also triggered the accumulation of the cytokinin *trans*-zeatin by 2.2–5.1 × in pepper leaves, compared to control.

Purine metabolites also increased following the microbial treatments. At 43 and 131 DAT, we observed sharp increases in the guanosine (2.7 × and 8.7 ×, respectively) and N6-threonylcarbamoyladenosine (3- and 7.8-fold, respectively) levels following microbial inoculation. Similar trends could be observed for DAT, NAD, and FAD at both 43 and 131 DAT, with increases by 1.5–4.4 × in biostimulant-treated plants.

## Discussion

There is a growing interest in the use of beneficial microbial inoculants such as AMF, *Trichoderma* spp., and PGPR in horticulture as they have multiple beneficial effects on crops (López-Bucio et al., 2015; Rouphael et al., 2015). Similarly, to other studies (Conversa et al., 2013; Colla et al., 2015; Bakr et al., 2018), microbial inoculation of pepper plants was effective to raise significantly the mycorrhizal root colonization and the *Trichoderma* population in the soil rhizosphere.

In the present study, we observed an increase of early and total crop yield, compared to uninoculated plants. Colla et al. (2015) reported that compared with uninoculated field-grown zucchini plants, those supplied with live AMF *G. intraradices* and *T. atroviride* inocula presented with greater early and total yields. Similarly, in two field experiments, Ortas (2019) reported that mycorrhizal inoculation increased yield of the tomatoes, green peppers and eggplants and P and Zn uptake in comparison with uninoculated plants. In the current experiment the total yield increase resulting from inoculation of sweet pepper plants with AMF and *Trichoderma koningi* was higher (24%) than the value (18%) reported by Ombódi et al. (2019) using an inoculum containing six different arbuscular mycorrhizal species under unheated greenhouse conditions and the value (12.7%) recorded by Almaca et al. (2013) using an inoculum containing *Glomus mosseae* and *G. etunicatum* under field conditions. The above differences in pepper yield response could be attributed to the different mycorrhizal species used in the trials and the addition of *Trichoderma koningi* in the current experiment. Co-inoculation of *Trichoderma* spp. and AMF have been found to promote growth and plant development of several vegetable crops more than inoculation using only *Trichoderma* spp. or AMF (Colla et al., 2015). Moreover, similarly to the trial reported by Ombódi et al. (2019), we observed a better yield response of pepper to mycorrhizal inoculation (+66% in the second fruit harvest made on 9 January—174 DAT) when the microclimate conditions for plant growth were suboptimal (low light and temperature occurring during January). Finally, in the current experiment the total yield increases induced by inoculation with AMF and *Trichoderma koningii* were due to both higher fruit number and mean fruit weight whereas in the trial of Ombódi et al. (2019) the yield increases were mostly due to higher number of fruits. The above findings indicate a reduced activity of indigenous arbuscular mycorrhizal fungi and *Trichoderma* spp. in enhancing crop productivity in comparison with exogenous selected arbuscular mycorrhizal fungi and *Trichoderma* species under field conditions. Similarly, Ombódi et al. (2019) reported that inoculation of pepper plants at transplanting with a commercial product containing six different arbuscular mycorrhizal species was able to enhance mycorrhizal root colonization, leaf chlorophyll content (SPAD index) and fruit yield in comparison with naturally occurring mycorrhizal fungi in untreated control. The above findings may be explained by the depression of native mycorrhizal fungi in horticultural production systems caused by the frequent soil tillage and the overuse of chemical inputs. Under these conditions, AMF inoculation may compensate for the loss of indigenous microbial communities to support plant growth (Yu et al., 2020). The results of the current experiment proved that exogenously-applied beneficial fungi such as AMF and *Trichoderma koningi* act as phytostimulation agents and improve plant nutrient uptake. The phytostimulation efficacy of beneficial fungi is explained by complex signal exchange and crosstalk between the host plants and the microorganisms affecting phytohormone balance and plant metabolism (Sbrana et al., 2017). Metabolomics helps elucidate the metabolic pathways and processes involved in plant-microbe interactions. Growth stage has a hierarchically strong effect on the leaf metabolome. Nevertheless, microbial biostimulants significantly alter the metabolome such that it is readily distinguishable from the control. The microbial treatments elicited several processes related to plant secondary metabolism. Microbial-based biostimulants promote the accumulation of different classes of secondary metabolites and phospholipids.

Plant responses to microbial-based biostimulants involved the modulation of phytohormone network. Treatments with beneficial fungi alter auxins, cytokinins, and gibberellins. Modification of the hormone profile may be associated to the yield increases we observed. Several studies demonstrated that microbial biostimulants promote yield by changing the phytohormone balance, increasing nutrient availability and uptake, and enhancing abiotic stress tolerance (Rouphael et al., 2015; Saia et al., 2020). Certain putative mechanisms for the biostimulant activity of microbial-based inoculant (AMF + *Trichoderma*) in pepper have been proposed. Microbial-based inoculants promote root biomass, length, density, and branching, in turn increasing macronutrient and micronutrient uptake and boosting crop productivity. They also regulate key phytohormones such as gibberellins, cytokinins, and auxins (López-Bucio et al., 2015; Rouphael et al., 2015).

Gibberellins are diterpenoid phytohormones that regulate plant development, flowering, and senescence (Shu et al., 2018). In response to microbial-based inoculant treatment, gibberellins precursors increased. Although the precursor gibberellin A20 was recently linked to increased yields in maize (Tucker et al., 2019), the concurrent increase in auxins we observed (i.e., hormones upregulating the genes encoding 2-oxidases) suggests the promotion of gibberellins catabolism (Frigerio et al., 2006). Indeed, the coordination between gibberellins biosynthesis (mediated by 20- and 3-oxidases) and their 2-oxidases inactivation affects pollination and fruit set in tomato (Serrani et al., 2007). On the other hand, auxins and gibberellins overlap in terms of root growth and fruit set regulation (Bermejo et al., 2018). The microbial-based biostimulant also increased the accumulation of *trans*-zeatin; cytokinins interact with auxins to fine-tune root and shoot development. *Trans*-zeatin modulates meristem activity and mediates plant responses to variable extrinsic factors such as abiotic stress (Werner and Schmülling, 2009).

The modulation of plant signaling compounds in response to the microbial-based biostimulant treatment also involved membrane lipids. Phospholipids are plasma membrane components that play important roles in cell signaling, membrane trafficking, and apoptosis (Xue et al., 2009). The microbial-based biostimulant treatment changed the phospholipids profile. It altered 20 foliar metabolites at 43 DAT (vegetative stage) and 31 foliar metabolites at 131 DAT (reproductive stage). Lysophospholipids release calcium from the endoplasmic reticulum, promote cell division and inhibit apoptosis (Ye, 2008; Hou et al., 2016).

The microbial treatment also modulated secondary metabolism, i.e., a set of processes often altered in response to plant interactions with the environment, including agronomic practices and plant-microbe interactions (Yang et al., 2018). In our experiments, plant responses to microbial biostimulants entail the coordinated modulation of several unrelated pathways.

The carotenoids vitamin A and α-carotene increased following the microbial treatment. Carotenoids absorb light energy, participate in photosynthesis, protect plants against oxidative damage, and are precursors of visual pigment chromophores and volatile apocarotenoids that attract pollinators (Heath et al., 2013; Sun et al., 2018). Moreover, they are involved in plant responses to abiotic stresses and plant-microbe interactions (Felemban et al., 2019). Among carotenoids, blumenols also accumulated in the biostimulant-treated plants. Noteworthy, they are reported to accumulate in roots and shoots of mycorrhized plants and have been proposed as markers of arbuscular mycorrhizal fungi colonization (Wang et al., 2018). However, their functions in processes other than allelopathy are still unknown. Their levels are strongly correlated with the degree of mycorrhization (Fester et al., 2002). Concerning saponins, they are constitutively produced in plants and comprise part of plant defense, having both antifungal and antifeedant activity. Though they are generally associated with pathogenesis, it was reported that saponins may participate in mutualistic relationships among plants, rhizobacteria, and mycorrhizae (Szakiel et al., 2011). Despite not focusing on root metabolome (where such mutualistic associations take place), our results indicate that saponins may also be involved in aboveground response to microbial inoculation with the biostimulants.

Compared to control, plants subjected to the microbial treatments presented higher levels of phenolic compounds. Phenolic metabolites are essential for lignin and pigment biosynthesis and participate in plant responses to pathogens and external stimuli (Bhattacharya et al., 2010). Mycorrhizae elicit phenolic biosynthesis in other plant species (Baslam and Goicoechea, 2012; Jugran et al., 2015). They also trigger plant defense against abiotic and biotic stresses and improve nutrient availability and use efficiency (Sharma and Sharma, 2017). Phenolics are associated with plant defense mechanisms. Flavones may protect plants from both biotic and abiotic stress (Martinez et al., 2016). Lignans have high antioxidant activity (Hu et al., 2007; Durazzo et al., 2013). Compared with the uninoculated, the gibbilimbol B level was 1.5 × and 4.2 × higher at 43 and 131 DAT sampling dates, respectively, in the inoculated plants. Gibbilimbol B was reported to have fungicidal activity against *Fusarium oxysporum* f. sp. *dianthi*. Coumarin upregulation is related to iron nutrition (Curie and Mari, 2017), allelochemistry (Niro et al., 2016), and abiotic stress tolerance (Saleh and Madany, 2015) in plants. Plant coumarins may influence the shape of the root microbiome (Voges et al., 2019).

Relative to the control, the levels of several purines were altered in the plants treated with the microbial biostimulant here. Several studies have focused on the effects of increased levels of adenosine and purines. These compounds are recycled by the so-called “salvage pathway” (Ashihara et al., 2018). Nicotinamide adenine dinucleotide (NAD) and flavin adenine dinucleotide (FAD) are reducing equivalent exchange cofactors that participate in several redox reactions.

Overall, our metabolomics study revealed that microbial biostimulant treatment had two major effects on pepper. First, the biostimulant modulated the phytohormone profile and phospholipid signaling in the plants. Next, it altered various secondary metabolic processes involving saponins, blumenols, carotenoids, and phenolic compounds. Phytohormones and biochemical messengers are associated with various metabolic processes (Ashihara et al., 2018) and might account for the observed biostimulant-mediated increases in crop productivity. Although it is difficult to ascribe the increased yield to few/some specific compounds, we can postulate that the altered balance of hormone profile may have played a pivotal role in fruit setting and development. Indeed, it is well recognized that yields are tightly connected to hormones profile, with an important role actually played by auxins (that increased in our experiments) (An et al., 2020). On the other hand, the involvement of phytohormones in the connection between beneficial microbes and plant productivity has already been postulated (Bhattacharyya and Jha, 2012). Comparatively, much less is known to date regarding the signaling related to membrane lipids, and future research is advisable on this topic.

The secondary metabolites modulated by biostimulant treatment have numerous positive influences on plant productivity, such as the enhancement of nutrient uptake and assimilation and biotic and abiotic stress tolerance. The elicitation of secondary metabolism by plant beneficial microbes deserves further investigation in terms of abiotic stress tolerance and induced systemic response (ISR) induction. Noteworthy, looking at food nutritional traits, carotenoids and phenolics improve quality and promote health in many fruits, including pepper. Thus, the microbial biostimulant treatments applied here could have nutritional implications as well.

## Conclusion

Recent scientific investigations have focused on improving sustainable farming practices that stabilize yield under optimal and suboptimal conditions and comply with changing legislation regarding the application of low-input agrochemicals. Microbial-based biostimulants (for example, AMF and/or *Trichoderma*) may sustainably enhance crop productivity. Our greenhouse experiment on pepper confirmed that inoculation with a combination of AMF and *Trichoderma koningii* TK7 increased marketable fruit yield by 23.7% relative to that of the untreated control. Metabolomics analysis revealed that the biostimulant treatment reprogrammed the leaf metabolome at the vegetative and reproductive stages. Likely, several biochemical processes underly the observed increase in fruit yield. Here, we showed that the biostimulant modulated the phytohormone profile and elicited secondary metabolism. Specifically, the microbial-based biostimulant upregulated compounds such as carotenoids, saponins, and phenolics that participate in plant nutrition, defense, and stress response. The results of the present study confirm that biostimulant amendments improved the plant health status since the vegetative stage, favoring stable increases in fruit yield. This leads the way toward future investigations into their effects on plants under challenging conditions such as abiotic and biotic stress, environmental perturbations, and physicochemical imbalances.

## Data Availability Statement

The original contributions presented in the study are included in the article/Supplementary Material, further inquiries can be directed to the corresponding author.

## Author Contributions

PB, GC, YR, VC, MC, and LL designed the experiment. GC, MC, YR, and GE measured and made the interpretation of agronomical data. PB and BL acquired the metabolomics and qPCR data. PB, BM-M, and LL analyzed the metabolomics data. All authors discussed the results and contributed to the final manuscript.

## Conflict of Interest

VC and GE were employed by the company Atens SL. BL and PB were employed by the laboratory NGAlab. The remaining authors declare that the research was conducted in the absence of any commercial or financial relationships that could be construed as a potential conflict of interest.
